# Evolution of public health policy on healthcare self-management: the case of Ontario, Canada

**DOI:** 10.1186/s12913-023-09191-3

**Published:** 2023-03-14

**Authors:** Amélie Gauthier-Beaupré, Craig Kuziemsky, Bruno J. Battistini, Jeffrey W. Jutai

**Affiliations:** 1 Faculty of Health Sciences, University of Ottawa, Ottawa, Ontario Canada; 2 Office of Research Services and School of Business, MacEwan University, Edmonton, Alberta Canada

**Keywords:** Public health policy, Self-management, Healthcare, Health policy analysis, Archival research

## Abstract

**Background:**

As people live longer, they are at increased risk for chronic diseases and disability. Self-management is a strategy to improve health outcomes and quality of life of those who engage in it. This study sought to gain a better understanding of the factors, including digital technology, that affect public health policy on self-management through an analysis of government policy in the most populous and multicultural province in Canada: Ontario. The overarching question guiding the study was: *What factors have influenced the development of healthcare self-management policies over time?*

**Methods:**

Archival research methods, combining document review and evaluation, were used to collect data from policy documents published in Ontario. The documents were analyzed using the READ approach, evaluated using a data extraction table, and synthesized into themes using the model for health policy analysis.

**Results:**

Between January 1, 1985, and May 5, 2022, 72 policy documents on self-management of health were retrieved from databases, archives, and grey literature. Their contents largely focussed on self-management of general chronic conditions, while 47% (*n* = 18/72) mention diabetes, and 3% (*n* = 2/72) focussed solely on older adults. Digital technologies were mentioned and were viewed as tools to support self-management in the context of healthcare delivery and enhancing healthcare infrastructure (i.e., telehealth or software in healthcare settings). The actors involved in the policy document creation included mostly Ontario government agencies and departments, and sometimes expert organizations, community groups and engaged stakeholders. The results suggest that several factors including pressures on the healthcare system, hybrid top-down and bottom-up policymaking, and political context have influenced the nature and implementation timing of self-management policy in Ontario.

**Conclusions:**

The policy documents on self-management of health reveal a positive evolution of the content discussed over time. The changes were shaped by an evolving context, both from a health and political perspective, within a dynamic system of interactions between actors. This research helps understand the factors that have shaped changes and suggests that a critical evidence-based approach on public health policy is needed in understanding processes involved in the development of healthcare self-management policies from the perspective of a democratic governing system.

**Supplementary Information:**

The online version contains supplementary material available at 10.1186/s12913-023-09191-3.

## Background

Life expectancy continues to increase globally, leading to an aging population that will crest in the next generation. Between 2000 and 2019, there was an increase in global life expectancy from 66.8 years to 73.4 years, respectively [1]. In Canada, the life expectancy increased to reach an 86-year-old average in either sex [2]. For many, it may afford increased time to spend with loved ones and more time to participate in various social and physical activities. However, the increase in life expectancies also comes with an increased risk of developing chronic diseases and comorbidities that may result in disability [2]. Living with chronic diseases and disability can lead to various disabling conditions that often trigger some level of healthcare self-management. Van de Velde et al. [3] define self-management as “the intrinsically controlled ability of an active, responsible, informed, and autonomous individual to live with the medical, role and emotional consequences of his chronic condition(s) in partnership with his social network and the healthcare provider(s)” (p.10). Chronic disease self-management is an important aspect of tertiary prevention, whose goal is to achieve a return to maximal function [4], and has only recently become part of urgent public health policy. Addressing self-management of disease promotes healthy living and well-being, which is one of the United Nations’ sustainable development goals [5], endorsed by Canada who has committed to advancing these goals [6].

Self-management can empower older adults to overcome barriers of an overwhelmed and underfinanced healthcare and social system, and leave them better equipped to face challenges in their everyday lives. Interventions to promote self-management, by developing abilities of patients (i.e., education, training and support), have shown to improve health outcomes and reduce healthcare utilization [7]. Conversely, this may reduce experiences of disability that these individuals would otherwise encounter which would make them – to some degree—stronger. Similarly, self-management of disability involves learning to live amidst the disability and forcing to find solutions to increase quality of life while still having to deal with everyday challenges of the disability. By self-managing diseases and disabilities, individuals can enhance their sense of autonomy and dignity, and thereby promote their mental and physical well-being [8]. As an enabler to self-management, digital technology can support activities such as exercising, healthy eating, medication management, monitoring of signs and symptoms, and problem-solving (in cases of distress for example) [9–12].

For Canadians, opportunities to become involved in self-management are numerous and often supported via programming such as adaptations of the Stanford Chronic Disease Self-Management Program [13]. For example, several provinces integrate the Stanford Chronic Disease Self-Management Program into community-based programs or deliver it through local health authorities [14]. While self-management support programs exist, they are not fully integrated within the healthcare system because healthcare providers have limited knowledge about their availability or how to refer their patients to them [15], which limits their effectiveness and ability to meaningfully contribute to improving the quality of life for older adults. Through the years, there have been several attempts by provinces and territories (editor’s note: Canada has 13 separate healthcare systems) to better integrate self-management into healthcare systems and in the lives of Canadians by developing policy actions on the issue, but research has shown that efforts are either disease-specific or embedded in population-wide approaches [14]. Uneven and inequitable implementation of self-management programming and supports limits the impact and reach of such efforts. For example, a focus placed specifically on diabetes management, may have limited impact for those who need to self-manage other chronic conditions or disabilities and functional limitations that may be linked to advanced age. In addition, policies that are focussed on the general population may have limited impact for certain segments of the population, such as older adults, that may have special concerns, needs and sets of difficulties in performing daily activities. This focus and perspective points to larger issues with the processes of policymaking regarding self-management in Canada. To address these issues, we need to undertake an in-dept scan of current and historical policies to understand how a variety of approaches to policymaking came about and to identify which factors have led to advancements (or not) in the area. As per the model for health policy analysis [16], several contextual elements (such as political regimes) and many diverse stakeholders influence how a policy is implemented into society. For example, elements of context such as increased concerns with rising cases for chronic conditions, the political or economic system in a country at a specific time, or major societal events (e.g., the period studied here, COVID-19 pandemic) can all shape the evolution of policies on health self-management. The agenda of the actors involved in the policy creation could also influence the content and way in which policies are developed and implemented.

To develop effective policies on self-management that improve the quality of life for older adults with chronic diseases, developed comorbidities and disability, there is a need to understand why and how governments have undertaken policymaking on those needs. For the purpose of this analysis, Ontario will be used as a case study because it has worked to advance health promotion and prevention initiatives for as long as 35 years [17]. This paper examines the evolution of policies for self-management of health in Ontario to understand how the content has evolved based on context and actors involved in policy creation.

The research question for our study was: *What factors have influenced the development of healthcare self-management policies over time?*

Sub-questions:

1. How have policies and policy-related documents on self-management of health evolved over time in terms of their content and timing of major events and political timing in Ontario?
2. What elements of context have influenced policies on self-management of health in Ontario?
3. What actors were involved and how did the actors frame health self-management in the creation of policies in Ontario?

## Methods

### Methodological approach

The study was conducted using the READ approach to document analysis as it sets out a series of systematic procedures to gather, review and evaluate health policy documents [18]. The READ approach was used in previous research to ensure rigour in analyzing the health policy documents [18, 19]. The four steps in this approach were developed to ensure a rigorous process throughout document analysis, and include: 1) readying the materials, 2) extracting the data, 3) analyzing the data, and 4) distilling the findings [18]. The four steps of the READ approach are described below in how they were applied to this study.

While this study follows a rigorous process for collecting, analyzing, interpreting, and presenting the findings, it is positioned and co-constructed with the author’s view and perceptions of the documents. For example, the authors kept reflexive notes as they were coding the documents. It was noted that authors had a particular interest in the role of technology in supporting self-management. As such, they have added a code about technology to document the role of technology to support self-management as discussed in policy documents and this addition is also reflected in the findings of the study.

#### Step 1: readying the materials

##### Defining policy documents

In this study, archival research was used to identify and document the evolution of self-management policy in Ontario. Initially, policy documents were defined as a “formal statement that defines priorities for action, goals and strategies, as well as accountabilities of involved actors and allocation of resources” (p.94) [20]. While we were able to retrieve a few policy documents as per the definition, we decided to broaden our scope to other policy-related documents that were relevant to the topic of interest. More broadly, documents were included in this study (i) if they discussed self-management, (ii) if it was either a legislative document (including a policy), a strategic or action plan, a report (including environmental scans), an evidence brief, a set of guidelines or recommendations, a memo, a news media release, a fact sheet, or a framework for action, and (iii) if it was developed and published by or with a department of the Ontario Government.

##### Collection of policy documents

The search strategy was developed with the help from an information specialist at the University of Ottawa. A diverse set of platforms were included to ensure a comprehensive identification and retrieval of relevant policy documents (Additional file 1). The following sources were searched to locate relevant policy documents between January 1, 1985, and May 5, 2022: 1) the Archives of Ontario and Legislative Library of Ontario platforms to get direct access to government archives; 2) the Government of Ontario webpages to identify documents that would not be indexed in the archive databases; and 3) health and policy-specific databases to identify supplemental policy-related documents that were relevant to self-management of health. The Health Systems Evidence (HSE) repository was also verified to retrieve any additional materials not found in the other platforms and databases.

1. For the search in the Archives of Ontario and Legislative Library of Ontario, we obtained documents that discussed self-management in the context of health and healthcare. We established a search approach with a librarian from the Archives of Ontario to ensure that we accessed all relevant available collections and gathered a comprehensive set of documents relating to self-management. The first author of this publication conducted the searches and identified the relevant documents. The documents were included if the titles and summaries discussed self-management in the context of health.
2. For the search in the government of Ontario website, we used a keyword search through various websites’ search engines. The keywords included “self-management”, “self-care”, “self-monitoring”, and “self-efficacy”. The search results were screened and reviewed by team members who identified results that discussed self-management in the context of health.
3. For the search in academic databases, 8 databases were selected based on their scope and the type of content that they include ensuring that they were likely to publish policy documents. They included CINAHL (EBSCO), EMBASE (OVID), ProQuest Politics Collection (ProQuest), Canadian Business and Current Affairs Database (ProQuest), Canadian Public Policy Collection (Scholars Portal Books, the Canadian Periodical Index (CPI.Q), Academic Search Complete (EBSCO) and the Government and Legislative Libraries Online Publications Portal (GALLOP). The search strategy included keywords and database-specific thesaurus words on self-management, disease and disability, and Ontario (Additional file 1).

**Fig. 1:**
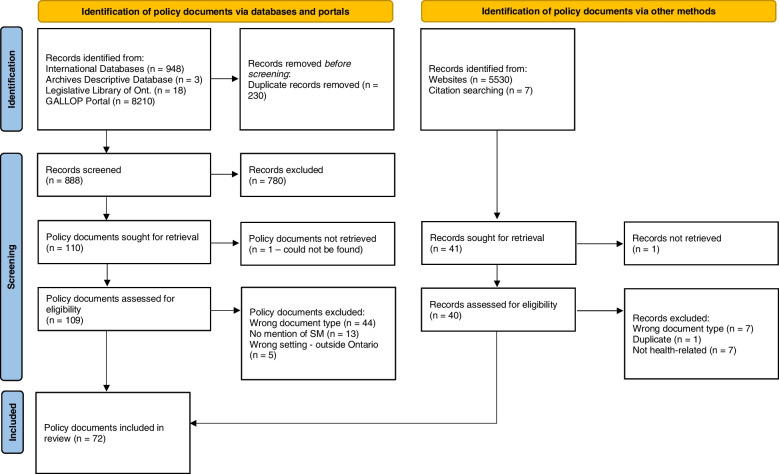
PRISMA flow diagram for archival research. *From: *Page MJ, McKenzie JE, Bossuyt PM, Boutron I, Hoffmann TC, Mulrow CD, et al. The PRISMA 2020 statement: an updated guideline for reporting systematic reviews. BMJ 2021;372:n71. doi: 10.1136/bmj.n71. For more information, visit: http://www.prisma-statement.org/

The policy documents were screened and assessed for eligibility (Fig. 1). They were included in the study if they were policy documents and developed by or with a department of the Ontario government.

#### Step 2: Extracting the data

##### Extraction, analysis and evaluation of policy documents

Data extraction, analysis and evaluation were performed using a modified version of the data extraction spreadsheet developed in a study on the Integrated Community Case Management of Childhood Illness Policies [21], as cited in Dalglish, S. L., Khalid, H. and McMahon, S. A. [18]. We used an excel spreadsheet where specific information about policy documents were recorded. These included descriptive information on the document authors, date of publication, type, and objective. A summary of the documents, information on the evidence cited and any relevant information on budget used in the creation of the document were also recorded. Additional details about the labels assigned are presented below.

#### Step 3: analyzing the data

**Fig. 2:**
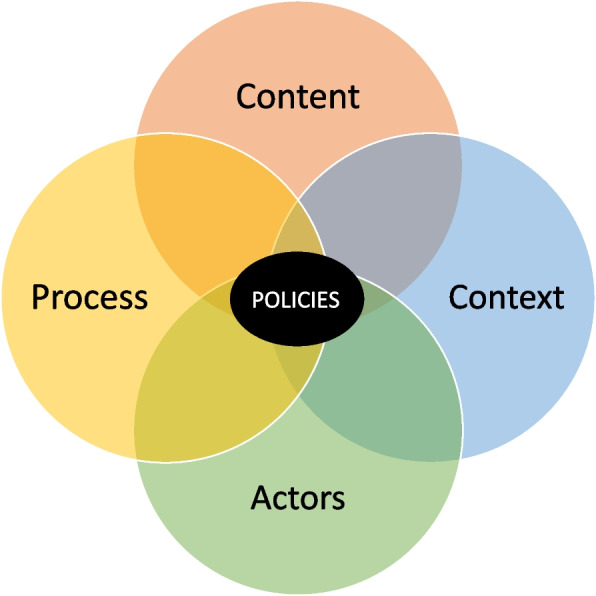
An adapted model for health policy analysis. This simple adapted analytical model for conducting policy analysis incorporates key concepts that need to be considered in the design of policies: Context, process, actors and content. Adapted from “Reforming the health sector in developing countries: The central role of policy analysis” by G. Walt and L. Gilson, 1994, *Health Policy and Planning, 9(4),* p. 354. Copyright 1994 by Oxford University Press

We used an adapted analytical model for health policy analysis (Fig. 2) to frame our analysis and evaluation of the policy documents. Walt and Gibson [16] position and define their model for policy analysis using examples from developed countries to amplify the detriment of one-sided policy models. However, the applicability and operationalisation of their model for policy analysis to developed countries is without contestation as it offers a holistic mechanism to assess various components of health policies. Walt and Gibson [16] suggested that, in the reform of health policies, too much of a focus is put on the content of the policies, ignoring that other dimensions such as context, process and actors all shape how changes in health policy occur. For example, external pressures from health advocacy/pressure groups, not-for-profits, charities, community organizations, personal experiences and interests with ideologies, cannot be ignored as they shape and create how policies are developed and implemented [22]. While the four dimensions of the model for health policy analysis are unique, they do not exist without pressures from others and are therefore interrelated [16]. Policies are highly influenced by the context within which they being developed without neglecting that there were, is, will ever be, a pervasion between the political and cultural factors of the moment [16]. In addition, the actors that are creating or influencing the policy shape policymaking from a variety of angles based on their own presumptions and assumptions. Pressures from actors advocating for change and those developing the policy are all influencing the end result of the policy. For this reason, we evaluated policy documents by reporting on the context, actors and content to better grasp and understand the political dimensions of policy changes. The process dimension, however, could not easily be identified solely from reading the documents since the process of policymaking is often not stated in these documents; therefore, it was omitted from the analysis. In addition, a review of documents did not allow to clearly understand and delineate the concrete influence of actors on content and context on actors as it requires a deeper understanding of the process for policy development. For this reason, actors are represented as a separate section in the results. By analyzing three dimensions of the policies (content, context and actors) and how they are outlined in the policy document, we will be better equipped to understand how healthcare self-management has been shaped in the context of Ontario.

Document analysis was used to interpret the documents for the three domains of interest. We used a deductive-inductive approach [23] where we identified preliminary codes based on the research questions and refined the codes as we were coding documents for the 3 domains until themes emerged and were defined [24]. The content of the documents was thematically analyzed by identifying key elements about self-management (frequency of mention, definition of self-management, approach to self-management [i.e., development of personal skills, collaboration between various actors, creation of educational resources, etc.]), identifying mentions of and the role of digital technology in self-management, and identifying the chronic diseases and disabilities of interest. For the context dimension, we have assigned contextual labels by identifying the political lead during document release, major events happening around the release date of the documents (i.e., COVID-19 pandemic), and whether there were significant financial implications around document release (i.e., budget funding in support of a policy). Specifically, the labels were: political lead, major events, and health-related budget. This step involved looking at supplementary files since contextual information was usually omitted or not disclosed in the policy documents. To do so, we searched the government of Ontario website for any information relating to political leads and budget, and searched media for major events at the time of the document release. Finally, the actors’ dimension was thematically analyzed using the following codes: implications from groups of actors (government sectors, community organizations, experts and engaged stakeholders), target groups for measures discussed in the policy, and intended users of the policy documents. During analysis, the data was sorted using multiple filters such as year of publication, political context and topics of interest to identify trends and emerging themes.

#### Step 4: distilling the findings

As a final step, the documents were organized into a timeline based on their date of release to better visualize the historical evolution of self-management in health-related policy and what political party was governing Ontario alongside the 35 years. This was done using the program XMind. The timeline supplements the sorting action that was performed in the late stages of analysis and allowed to visually represent some of the major findings.

## Results

A thorough search of archival policy documents on self-management in Ontario led to the retrieval of 72 documents. Documents included publicly available research and government reports (*n* = 25), news media (*n* = 16), information sheets (*n* = 12), frameworks and policies (*n* = 6), and webpages (*n* = 13). Descriptive information was gathered and organized in an excel spreadsheet (as demonstrated in Table 1) to better represent the diversity and characteristics of documents retrieved.

**Table 1:** Example data extraction of policy documents

**Document title**	**Date of publication**	**Authors**	**Document type**	**Document type and objective**
Directions from a Local Scan: Self-Management and Empowering the Person Living with Diabetes in the North Simcoe Muskoka Local Health Integration Network (LHIN)	November 2009	North Simcoe Muskoka LHIN	Report	Report summarizing local health status on chronic conditions and findings from a report completed by a member of the North Simcoe Muskoka Chronic Disease Prevention and Management Regional Action Group
Guide to Chronic Disease Management and Prevention	September 27, 2005	Family Health Teams, Ministry of Health and Long-Term Care	Guidelines/ Recommendations	This guide has been developed to assist groups that are forming Family Health Teams to plan chronic disease management and prevention programs for their patients. The guide is intended as a companion to the Guide to Strategic and Program Planning, which provides an overview of the strategic and program planning process
We're here to help you live well with diabetes	July 2010	Ministry of Health and Long-Term Care	Fact Sheet	Fact sheet with information about how to manage diabetes and the supports available to do so
Ontario's Action Plan to Transform Healthcare in London	March 9, 2012	Ministry of Health and Long-Term Care	News media	News release announcing the Action Plan for Health Care in Ontario specific to London, Ontario

Document analysis allowed researchers to identify several common themes for content, actors, and context. While results are reported as distinct from one another, the section on the context shows how all components of the model for health policy analysis are intertwined and influence one another.

### Content

The policy documents reveal important characteristics about focus areas and approaches to policymaking on self-management of health. Several themes identified by the researchers are described below.

#### Singular disease focus

The entirety of documents retrieved focussed on the management and care for chronic diseases. The documents focussed on sharing details about resources available in the community, promoting programs and services, providing evidence on chronic disease management, or underlining guidance, recommendations and frameworks to support effective chronic disease management in Ontario. The scope of the policy documents varied with some focussing on a single chronic condition and others discussed chronic conditions generally. The chronic condition that was the focus for most of the policy documents was diabetes (*n* = 21; 29%). In total, diabetes was mentioned in close to half of all policy documents on self-management (*n* = 34; 47%). Other documents were either targeting the whole population, specific to people living with chronic diseases (group as a whole) or specific to people living with other conditions (i.e., stroke, asthma, chronic kidney disease, chronic pain, chronic obstructive pulmonary disease (COPD) and heart failure, plaque psoriasis, hip and knee replacement) (Table 2). In addition to focussing on diverse chronic conditions, 11 documents were specific to people of certain age groups (children and youth (*n* = 9) or older adults (*n* = 2)) and 11 documents were specific to certain areas of the province (i.e., people living in a Southwest Local Health Integration Network (LHIN) (*n* = 1)).

**Table 2:** Target population group within policy documents on self-management

**Target population group**	**Number (n)**
All	18
People living with chronic diseases	22
People living with diabetes	21
People who have had a stroke	2
People living with chronic kidney disease	2
People living with COPD or who have had heart failure	2
People who have had hip and knee replacement	1
People living with asthma	1
People living with plaque psoriasis	1
People living with chronic pain	2
TOTAL	72

#### Central role of individuals with chronic diseases

The concept of self-management evoked in the policy documents was mainly related to the personal skills that people living with chronic conditions should have to be able to support their daily life, sometimes ignoring the role of family and caregivers. There was a tendency to put self-management as the responsibility of the one with a chronic condition but with great emphasis on self-management supports (i.e., community supports and healthcare provider support). These included providing the ability for people with chronic diseases to receive adequate training, education materials, and information resources on how to effectively manage their condition. The role of healthcare professionals (such as primary care providers) was also mentioned as being essential and favouring effective and purposeful self-management by people living with chronic conditions. The central responsibility, however, remained mostly on the individual living with a chronic condition, with the support of their family/friends/community entourage.

#### Lack of digital technology integration

Considering the increase and evolution in the use of digital technology for healthcare, we retrieved specific details on the mentions of technology within the policy documents. While sometimes mentioned, the documents that did include digital technology did not always effectively link self-management with technology. They were mentioned sporadically and mostly in the presentation of specific initiatives and programs, and in relation to its benefits for enhancing effectiveness in healthcare settings. For example, a specific technology was promoted in the showcase of innovative programming that included a technological component [25]. Such technology was also mentioned in a review of best practices as one of the best mechanisms to influence health risk behaviours [26]. It was also re-acquired by several news media outlets and other documents, such as a newsletter about Ontario Diabetes Strategy, where they mentioned that there would be support for the adoption of new information technologies [27]. Most mentions about digital technologies were in the “Preventing and Managing Chronic Diseases: Ontario’s Framework” from May 2007 [28]. In this document from the Ministry of Health and Long-Term care, technology was mentioned for its opportunity, as a connected digital tool, to allow for telehealth in clinical settings and where providers have increased access to software to support decision-making [28].

#### Absence of research evidence in policy

Research evidence, in the form of citations of peer-reviewed scientific literature, was noticeably lacking within the policy documents. The documents that cited research evidence were in the form of guidelines (*n* = 9; 13%), recommendations (*n* = 9; 13%), or reports (*n* = 25; 35%). The evidence cited included statistics on chronic diseases in Ontario and in specific regions of the province. Some news media did also include some research evidence. Similarly, few documents mentioned the use of theories and models to frame their narratives. Some of the well-known Canadian models and frameworks cited included the Ottawa Charter for Health Promotion [29], Ontario’s Chronic Disease Prevention and Management Framework [28] and British Columbia’s Expanded Care Model [30]. In addition, certain documents mentioned international models and approaches such as the Chronic Care Model [31] and the Stanford Chronic Disease Self-Management Program [13]. Some documents also referred to smaller-scale programs implemented in other countries around the world.

### Actors

Actors of policy documents can be viewed as two-fold: those who develop and implement the policy and those who will be the beneficiaries or end users of the policy. In both cases, document analysis revealed the diversity of actors involved or impacted by the policy documents. Key themes identified by the researchers are described below.

#### Range of actors involved in policy development

The lead authors for the policy documents were numerous and varied between different Ontario government ministries, agencies, LHINs, and research centres. Close to three quarters of all documents retrieved were led and authored by varying structures of the Ontario government. The former Ministry of Health and Long-Term Care (now separate ministries) led one third of all publications that were authored by the Government of Ontario. Other Government of Ontario-lead policy documents were authored by Health Quality Ontario, specific LHINs, the Ministry of Children, Community and Social Services or the Ontario government generally without mention of specific departments or agencies. Our review identified that in addition to the lead authors, actors from a variety of sectors were consulted during document creation and contributed to the document. They included large not-for-profit organizations, community organizations and engaged individual stakeholders (advocates or researchers). For large organizations, those involved had specific knowledge about the health issue being discussed in the policy document. For example, for policy documents on diabetes, the Canadian Diabetes Association was usually cited as a contributor in the document. For community organizations, their role in the development of the document was mainly in a consultation role where they were able to share best practices from work happening in their communities. Finally, our review of the documents also indicated that individuals, as engaged stakeholders, were involved in a consultative role where they shared their real-life opinions and dialogue with others on a specific topic of interest. However, some documents did not explicitly cite what actors were involved in the document development.

#### General public as end users

The intended users of the policy documents were numerous. Some policy documents were specifically intended for policymakers and researchers while most of them targeted the general public (people living in Ontario), with some policy documents targeting specific groups like people living with diabetes in Ontario or healthcare providers in Ontario. These policy documents were mainly for information-sharing purposes and provided information about work being done in collaboration with and by the Government of Ontario. This was the case for much of the documents pertaining to the Ontario Diabetes Strategy where there was a significant focus on promotting good practices and new developments (i.e., programming).

### Timeline and evolution over time

**Fig. 3:**
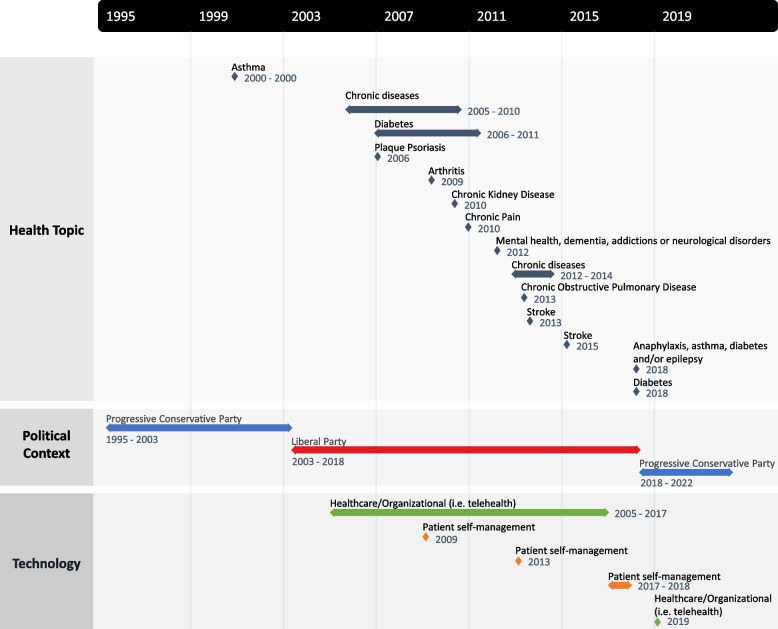
Timeline of policy documents per health topic, political lead, and digital technology considerations

While many policy documents on healthcare self-management have been published, content has changed significantly over time. Using the health policy model [16], we were able to identify some contextual factors that may have influenced the content and focus of these documents. In a timeline, three of the four major components of the model for health policy analysis (content, context, and actors) were evaluated (Fig. 3).

In short, the first policy document on self-management was published in October 2000 and focussed on asthma. A change in focus occurred shortly after, where chronic diseases more generally became of greater importance. This remained constant throughout time but some specific conditions, such as diabetes, received attention at different moments in time because of how fast they were growing (i.e., 69% increase in 10 years for diabetes) and how they are associated to expensive healthcare costs [32]. Self-management, initially viewed as being the responsibility of the individual, changed through time and became the responsibility of a larger team where care and management were seen as a collaborative effort that involved several professionals, community services, and key tools such as technology. Finally, time has also presented a change in the collaborative nature of policymaking. There was a clear shift from top-down to a hybrid between top-down and bottom-up healthcare governance approach to policymaking.

### Context

Through an analysis of context, we have identified themes that help to explain the reforms that occurred in content and actors dimensions of self-management policies in Ontario. As mentioned by Walt and Gilson [16], the interplay between each dimension is critical due to the influence that one dimension puts on others (i.e., influences from actors on the content; influences from context on actors and content; etc.). To do so, we have conducted an evaluation of policy documents to identify influences from context on the different aspects. The contextual factors that have influenced policy development over time are described in more detail below.

#### Pressures on healthcare system: increasing burden of chronic diseases and shifting models of care

First, as mentioned above, October 2000 represents the date when the first policy document on self-management was published by the former Ministry of Health and Long-Term Care. This report from the chief medical officer of health focussed on illustrating the burden of asthma on Ontarians and placing asthma as an important public health concern in Ontario [33]. At that time, asthma was viewed as an important public health concern in Ontario especially for children and adults due to increased absenteeism in school and from work [33]. In that document, self-management was mentioned in one specific section and the emphasis was put on the need for individuals to have self-management plans to help them manage various aspects of their conditions such as symptoms [33]. The self-management strategies identified in this publication were specific to asthma and did not include mention of other chronic conditions with similar affects. The document creation was informed by a steering committee composed of individuals working in the Ministry of Health and Long Term Care, in local health units, in various healthcare associations, and included academic research scientists. Since the release of this first publication, publications on self-management changed in focus significantly. The focus went from asthma to chronic disease management and prevention, at-large, when the Ministry of Health and Long-Term care published a guide on chronic disease management and prevention five years later, in September 2005 [34]. This guidance document was developed to assist groups forming the initial Family Health Teams (FHTs) to plan chronic disease management and prevention programs for patients [34]. When analyzing the self-management components in the document, it became clear that an increased emphasis was placed on the need to have supportive healthcare systems that promote patients’ self-management. The policy documents even demonstrated how self-management roles and responsibilities changed from the being on the individual to becoming an interdisciplinary collaboration between various stakeholders that function in tandem to create a good functioning health management environment. For example, Ontario’s Framework for preventing and Managing Chronic Disease [28] showed a shift in focus toward developing a system that was collaborative and where interdisciplinary efforts were made to promote patient empowerment and increase patient education. The increase in care personalization movements across time [35], where patients are involved in their care through participatory medicine or patient empowerment initiatives, also helped to explain this shift in policy focus. In addition, supports in the form of information technologies have also been added as relevant tools for self-management around 2005. They were being promoted in various documents as effective tools to support some components of self-management such as being connected with professionals for consultations, but mostly its benefits aimed at more effective healthcare system management. Overall, results pointed to an evolving understanding that chronic disease management required an interdisciplinary approach that involves the individual, healthcare professionals, community supports, and innovative technologies which leads to patient empowerment and effective self-management.

#### Political context

Looking deeper at the political context, the first publication about chronic diseases self-management generally was a guide on chronic disease management and prevention published in September 2005 by a Conservative government in Ontario [34]. It was followed by several publications published under a Liberal government in Ontario until 2018. During the lead of the Liberal party in Ontario, healthcare budgets shifted from focussing on increasing investments in healthcare facilities [36] to targeting spending on FHT, and contributing investments in medical technologies and home care. Chronic disease management and prevention continued to be the focus in all publications under the Liberal government until the most recent one in 2019 [37]. However, diabetes specifically gained traction in many publications since 2006. Several reports, news media and information sheets have focussed largely on diabetes, its impact, its management, and promoting exemplar programming that address challenges in daily lives of people living with diabetes. This specific focus lines up with the development of the Ontario Diabetes Strategy in July 2008. In addition, other chronic diseases received attention in policy documents since 2009. They include plaque psoriasis, chronic pain, chronic kidney disease, dementia, COPD, stroke, heart failure and epilepsy. The context underlying choices of these conditions could be explained by increases in prevalence, death, and hospitalizations for some conditions (epilepsy, dementia, chronic kidney disease, cardiovascular diseases, and COPD), and spendings over 10.5 billion dollars annually in direct healthcare costs [38, 39]. Contextually, these also align with funding for public health including health promotion and prevention initiatives, as outlined in budget documents from 2005 onward under the lead of the Liberal party in Ontario.

#### Hybrid top-down and bottom-up policymaking: collaborative policy development

Looking specifically at policy development, the actors involved in document creation have somewhat changed over time. In the initial documents from 2000 to 2007, the contributors originated mainly from within the Government of Ontario (i.e., Ministry of Health and Long-Term Care, Ministry of Health Promotion, etc.). In the following years, however, the actors involved shifted and showed an engagement of smaller government organizations including specific LHINS, academic research centres, individual health experts, and associations from the community. Such consultations with stakeholders (individuals, community organizations and diverse government agencies or departments) remained constant thereafter. This indicates a trend toward using both bottom-up and top-down governance models and allows for better collaboration and partnership between relevant stakeholders. Some documents did not include information on who was consulted in the development of the documents while others provided detailed information.

Overall, several contextual factors impacted policies on health self-management in Ontario. There is a strong interplay between content, context and actor components of policymaking which shaped the result and how policies ultimately impacted those engaging in self-management of health. Factors that impacted policies on self-management of health in Ontario include pressures on healthcare systems, the political context, and hybrid policymaking.

## Discussion

An analysis of Ontario government policy documents about healthcare self-management from October 2000 to June 2019 identified the following key events. The first published document, led by the government of Ontario, focussed on asthma and proposed some tips on how individuals could better self-manage their condition. This document, which was published under the lead of a Conservative party in Ontario, placed individuals living with asthma at the forefront of self-management and as holding responsibility for doing so. In subsequent years and with a change to a Liberal government, the focus shifted to general chronic conditions. Not long after, in 2006, there was an increase in publications on self-management of diabetes more specifically. This specific focus aligned with the release of the Ontario Diabetes Strategy in 2008. From there, publications that focussed on general chronic diseases and diabetes self-management portrayed self-management as a holistic activity that involved various individuals and disciplines in the support system. In addition, leads and collaborators of the publications included people that were closer to the “ground” and from more regional and community backgrounds. Several other publications were released, in an ad hoc manner, on other chronic diseases, and around 2005, documents were starting to include mentions of information technologies as innovative tools to support self-management.

**Fig. 4:**
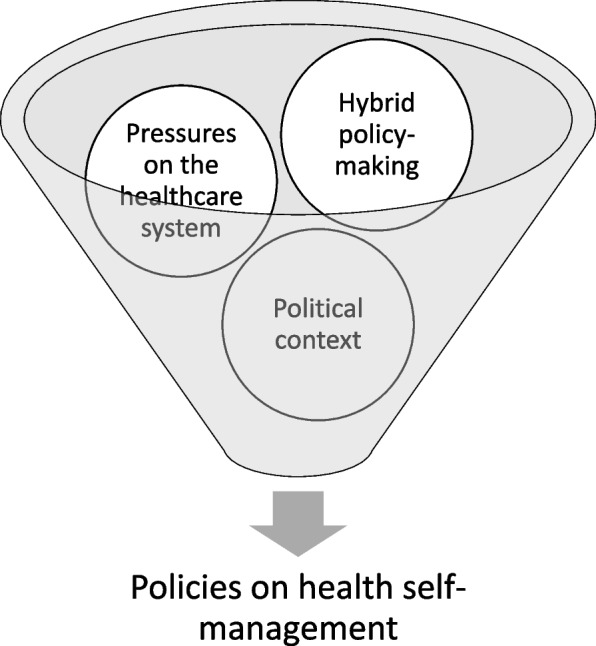
Factors that shape health self-management in Ontario

Factors that appeared and identified to be most influential on the nature and timing of these policies were pressures on the healthcare system and healthcare transformation, hybrid top-down and bottom-up policy development, and political context (Fig. 4).

Changes in the content within the policy documents were linked to pressures that impacted the healthcare system. With a focus on chronic diseases and projections that chronic diseases would account for millions of deaths around the world [40], Ontario was no stranger to the increased burden of chronic diseases on the healthcare system. The focus of the Government of Ontario toward self-management of general chronic diseases demonstrated a better understanding about the magnitude and impact of chronic diseases on the healthcare system. Additionally, the increased number of publications that focussed on diabetes since 2006 related with the increase in cases of type 2 diabetes in developed countries [41]. In the early 2000s, Ontario even surpassed global rates reported by the World Health Organization (WHO) for the prevalence of diabetes [41], which amplified the need for government leaders to take action. For type 2 diabetes, where lifestyle and healthy living behaviours can fend its offset, action by public health leaders and governments were foreseeable and desirable. In 2008, the government released the “Ontario Diabetes Strategy” which aimed to prevent, manage and treat diabetes, and provided millions of dollars in investments over a four years [32]. This demonstrated that the focus of the content within the policy documents were recognized from real and existing pressures of diabetes on the healthcare system. Seventeen years after its implementation, however, diabetes care and management can still be improved by creating policies that effectively supports its self-management [42]. Throughout time, the focus of the system has evolved from a medicalized system where there was a predominant focus on the diagnosis and treatment of pathological and biological issues, to one that promotes greater autonomy, health promotion and population health strategies [43]. As demonstrated in the policy documents retrieved, patient empowerment and supportive environments became integral parts of the management of chronic diseases. The reviewed literature further demonstrated how patients could and should be seen as equals in the caring for their conditions where both healthcare professionals and patients are viewed as experts in their respective areas and cannot function without one another: healthcare professionals bridge gaps in health literacy while patients are the experts on a personal level [44]. As pointed out in a report from the World Health Organisation in 2002, “optimal [health] outcomes occur when a healthcare triad [(including patients, healthcare professionals and community supports)] is formed” (p. 7) [45]. In Ontario, these concepts of collaboration and patient empowerment were integrated in policies throughout time, which suggests a willingness of the policy system to evolve toward a more collaborative and personalized model of care. While pressures on the healthcare system (increasing burden of chronic diseases and shifting models of care) and transformation of healthcare have shaped policy development, innovation in technology seemed to have had limited effects. The early 2000s have marked significant advancements in the digital technological sphere where many devices such as smartphones, tablets, and social media were born and refined. However, policies on self-management during that time missed key developments that could have facilitated and supported self-management in a more connected way. As mentioned in a scoping review by Jacelon, Gibbs and Ridgway [46], significant work on technology to support self-management was being done all around the world. Some benefits of technology-supported self-management include enhancing the healthcare system and narrowing the distance between patients and healthcare professionals by allowing them to be connected to one another more easily and rapidly [46], and increasing competence and illness management for patients [47]. These benefits, however, may have been suppressed by limits in the technological infrastructure such as limited access to computers and internet during that period [46]. In Ontario, barriers of access to digital technological infrastructure (due to cost of technology and its infrastructure or fluctuating digital literacy) may help to explain the delay in having technologies that support self-management included in policies. Since the start of the present decade, Ontario made significant investments in digitalization, positioning technologies for self-management as having a much clearer role within the system. In addition, the recent COVID-19 pandemic has exacerbated the need to have technologies to support individuals who are managing chronic conditions. During the pandemic, technology has proven to allow for continued healthcare services, improve health outcomes, physical and mental health, and enhance social connectedness of many individuals who are managing their chronic conditions, including older adults [48–51]. Therefore, digital technology has now become an integral and ineluctable part in every policy on self-management.

The results of this study portray important changes in how policy were developed through time. First, when policies on health self-management were first developed in 2000, policy documents seemed to have taken more of a top-down approach where policymakers were the sole drivers of policy development. However, the shift toward a hybrid approach to policymaking that includes both a top-down complemented by bottom-up approach demonstrated a willingness to consider field experts and lived experience in policy development. As stated in work by Sabatier [52], bottom-up approaches involve a network of actors that are actually involved in the execution of policies and programs. Through ongoing consultations with lower levels of decisions-making (bottom-up), involving community organizations and expert stakeholders, novel policies become more adapted to the context of communities. A hybrid healthcare governance model can provide significant benefits and improvement to healthcare [53]. This hybrid model is demonstrated well in the Ontario Diabetes Strategy, where it is driven by the Ontario government but with working-level policymakers and external organizations are involved throughout. The documents demonstrated that much of the hands-on work of the Governemnt of Ontario for the Ontario Diabetes Strategy was developed and ran by regional and community-level organizations. Second, ways by which policies are developed are not only the reflection of who is consulted and involved, but also influenced by the political context. For most of the years during which self-management publications were released, the Ontario government was under the lead of a Liberal party. The in-depth analysis of the budgets from the Liberal government between 2003 and 2018 revealed that there were significant investments in health promotion and prevention programs. Funding in these areas demonstrated a willingness for a liberal government to support prevention and management initiatives which include self-management. In 2005, the Liberal government even established the "Ministry of Health Promotion" as a way to promote healthy choices and healthy lifestyles for Ontarians [54]. This political willingness to promote healthy lifestyles corroborated with increased policies on self-management of chronic diseases.

Finally, our analysis revealed extensive foci for self-management of chronic diseases generally. It became evident that while not all chronic diseases had received attention in policies in Ontario, the general concept of chronic diseases was an area of great interest. This has implications in that not all individuals would adequately benefit from supports in self-managing daily difficulties because policies and related services would take a more generic approach. Furthermore, self-management is never discussed in terms of disabilities or functional limitations related to aging. While disabilities and functional limitations related to aging may have different implications for individuals, similar strategies as the ones used in chronic disease self-management could support older adults living with disabilities or functional limitations to have an improved quality of life. Finally, limited discussions on the role of technologies in supporting self-management reveals that uptake of innovation to support self-management has been slow. This leaves room for the Ontario government to exploit new and effective avenues to improve self-management supports for Ontarians.

## Limitations

The study has several limitations that rely merely on the nature of the data retrieved. The data collected were policy documents published in online archival repositories, websites, or databases. While the documents retrieved offer a comprehensive overview of the policies on self-management and their evolution over time, they may not adequately portray the full picture for policymaking on the issue. Retrieval of such documents also came as a challenge which could have led to documents being missed. In certain cases, documents may have been in other formats and not available via online repositories.

Regarding the framework of analysis for this study, we have selected the approach which we believe would allow for a more comprehensive analysis of the documents. While many frameworks for analysis focus on evaluating the content of policies, they would omit critical components such as actors and context which all contribute to shaping policy development [16]. Unfortunately, the process with which policies have been implemented could not be analyzed in this study due to the nature of the data collected but other critical components of policy development (content, context and actors) were analyzed in detail. For actors and context components, our analytical approach included the retrieval of supplementary documentation (i.e. budget documents) and identifying critical authorship and consultations mechanisms which offers a limited view of the full historical policymaking approach. These limits will be addressed in future steps of the project which include consultations with current policymakers working in the field of self-management. For these reasons, Walt and Gilson’s [16] model for health policy analysis was selected for the analysis and evaluation of the data, and was the best suited to answer the research question.

## Future research

Future research should continue to document the evolution of self-management policy in Ontario and evaluate the effects of the factors explored in this study (shown in Fig. 4). In addition, future research could focus on evaluating the process for developing and implementing policies on self-management as this component could not be assessed from the retrieval of documents alone. This would require that other means of data (i.e., interview data) to complement the results found from documents. Finally, research that investigates present approaches for developing, implementing, and evaluating self-management policies could help to better understand current practices for supporting self-management from the perspective of the system more broadly, and offer a more updated and accurate picture of policymaking in the current context.

## Take-home messages

- Healthcare self-management is a concept that first appeared in Ontario policy documents in 2000.
- Healthcare self-management policies in Ontario have focussed largely on chronic diseases and diabetes, without consideration for people living with disabilities and functional limitations that come with age.
- Digital technology has received limited attention in policy documents for its potential to support self-management of chronic diseases while significant technological advancements have been made in this area.
- Several factors have been influential the evolution of self-management policies in Ontario including changes in pressures on the healthcare system and healthcare transformation, hybrid top-down and bottom-up policymaking, and the political context.

## Supplementary Information

**Additional file 1.** Detailed search strategy.

## Data Availability

The datasets used and/or analysed during the current study are available from the corresponding author on reasonable request.

## Abbreviations

HSE: Health Systems Evidence
LHIN: Local Health Integration Network
COPD: Chronic Obstructive Pulmonary Disease
FHTs: Family Health Teams
WHO: World Health Organization
NCE: Networks of Centres of Excellence

## References

[1] World Health Organization. GHE: Life expectancy and healthy life expectancy. 2019. Available from: https://www.who.int/data/gho/data/themes/mortality-and-global-health-estimates/ghe-life-expectancy-and-healthy-life-expectancy. [Cited 2022 Jun 13].

[2] Public Health Agency of Canada. Aging and chronic diseases: A profile of Canadian seniors. 2020 Dec p. 178. Available from: https://www.canada.ca/en/public-health/services/publications/diseases-conditions/aging-chronic-diseases-profile-canadian-seniors-executive-summary.html

[3] Van de Velde D, Zutter FD, Satink T, Costa U, Janquart S, Senn D (2019). Delineating the concept of self-management in chronic conditions: a concept analysis. BMJ Open.

[4] Lorig K (1996). Chronic Disease Self-Management: A Model for Tertiary Prevention. Am Behav Sci.

[5] United Nations. Goal 3: Ensure healthy lives and promote well-being for all at all ages [Internet]. United Nations Sustainable Development. Available from: https://www.un.org/sustainabledevelopment/health/. [Cited 2022 Jun 13].

[6] Government of Canada. Canada takes action on the 2030 Agenda and the Sustainable Development Goals. 2021. Available from: https://www.canada.ca/en/employment-social-development/programs/agenda-2030.html [Cited 2022 Jun 13].

[7] Panagioti M, Richardson G, Small N, Murray E, Rogers A, Kennedy A (2014). Self-management support interventions to reduce health care utilisation without compromising outcomes: a systematic review and meta-analysis. BMC Health Serv Res.

[8] Lorig K, Ritter PL, Pifer C, Werner P. Effectiveness of the Chronic Disease Self-Management Program for Persons with a Serious Mental Illness: A Translation Study. Community Mental Health Journal. 2014;50(1):96–103. 10.1007/s10597-013-9615-5. 10.1007/s10597-013-9615-5 23748554

[9] Choi W, Wang S, Lee Y, Oh H, Zheng Z (2020). A systematic review of mobile health technologies to support self-management of concurrent diabetes and hypertension. J Am Med Inform Assoc.

[10] Glasgow RE, Kurz D, King D, Dickman JM, Faber AJ, Halterman E (2012). Twelve-Month Outcomes of an Internet-Based Diabetes Self-Management Support Program. Patient Educ Couns.

[11] Hailey D, Roine R, Ohinmaa A (2002). Systematic review of evidence for the benefits of telemedicine. J Telemed Telecare.

[12] Hunt CW (2015). Technology and diabetes self-management: an integrative review. World J Diabetes.

[13] Office of Disease Prevention and Health Promotion. Self-Management Education: The Chronic Disease Self-Management Program. 2012. Available from: https://www.healthypeople.gov/2020/tools-resources/evidence-based-resource/self-management-education-chronic-disease-self [Cited 2022 Apr 12].

[14] Liddy C, Mill K (2014). An environmental scan of policies in support of chronic disease self-management in Canada. Chronic Dis Inj Can.

[15] Johnston SE, Liddy CE, Ives SM (2011). Self-management Support: A New Approach Still Anchored in an Old Model of Health Care. Can J Public Health.

[16] Walt G, Gilson L (1994). Reforming the health sector in developing countries: the central role of policy analysis. Health Policy Plan.

[17] Health Canada (1997). Health Promotion in Canada - A Case Study.

[18] Dalglish SL, Khalid H, McMahon SA (2021). Document analysis in health policy research: the READ approach. Health Policy Plan.

[19] Gage R, Leung W, Gurtner M, Reeder AI, McNoe BM, Signal L. Generating political priority for skin cancer primary prevention: A case study from Aotearoa New Zealand. Health Promot J Austr. 2021;n/a(n/a). Available from: http://onlinelibrary.wiley.com/doi/abs/10.1002/hpja.545. [Cited 2022 Apr 24]. 10.1002/hpja.545 34551173

[20] Bull FC, Bellew B, Schöppe S, Bauman AE (2004). Developments in National Physical Activity Policy: an international review and recommendations towards better practice. J Sci Med Sport.

[21] Bennett S, Dalglish SL, Juma PA, Rodríguez DC (2015). Altogether now…understanding the role of international organizations in iCCM policy transfer. Health Policy Plan.

[22] Brownson RC, Chriqui JF, Stamatakis KA (2009). Understanding Evidence-Based Public Health Policy. Am J Public Health.

[23] Fereday J, Muir-Cochrane E (2006). Demonstrating rigor using thematic analysis: a hybrid approach of inductive and deductive coding and theme development. Int J Qual Methods.

[24] Vaismoradi M, Turunen H, Bondas T (2013). Content analysis and thematic analysis: implications for conducting a qualitative descriptive study. Nurs Health Sci.

[25] Government of Ontario. Archived - Rural roadmap: The path forward for Ontario. ontario.ca. 2015. Available from: http://www.ontario.ca/page/rural-roadmap-path-forward-ontario. [Cited 2022 Apr 12].

[26] University Health Network Women’s Health Program. Literature Review - Best mechanisms to influence health risk behavior. Women’s Health Council, Ministry of Health & Long-term Care; 2000. p. 129.

[27] Tepper DJ, Government of Ontario, Ontario Ministry of Health and Long-Term Care (2009). Ontario’s Diabetes Strategy.

[28] Ministry of Health and Long-Term Care, Government of Ontario. Preventing and Managing Chronic Disease: Ontario’s Framework. 2007. Available from: https://www.health.gov.on.ca/en/pro/programs/cdpm/pdf/framework_full.pdf. [Cited 2021 Jul 28].

[29] World Health Organization, Health and Welfare Canada, Canadian Public Health Association. Ottawa Charter for Health Promotion. Ottawa, Ontario; 1987 p. 2. Available from: https://www.who.int/publications/i/item/ottawa-charter-for-health-promotion

[30] British Columbia Ministry of Health Planning (2003). Framework for a Provincial Chronic Disease Prevention Initiative.

[31] Wagner EH (1998). Chronic disease management: what will it take to improve care for chronic illness?. Eff Clin Pract.

[32] Government of Ontario. Ontario Launches Diabetes Strategy. news.ontario.ca. 2008. Available from: https://news.ontario.ca/en/release/1031/ontario-launches-diabetes-strategy. [Cited 2022 Apr 12].

[33] Ontario Ministry of Health and Long-Term Care. Taking Action on Asthma: Report of the Chief Medical Officer of Health. Ontario; 2000 Oct p. 16. Available from: https://www.health.gov.on.ca/en/common/ministry/publications/reports/asthma/asthma_e.pdf

[34] Ontario Ministry of Health and Long-Term Care. Guide to Chronic Disease Management and Prevention. Ontario; 2005 Sep p. 12. Available from: http://www.ontla.on.ca/library/repository/mon/14000/261463.pdf

[35] Karazivan P, Dumez V, Flora L, Pomey MP, Del Grande C, Ghadiri DP (2015). The Patient-as-Partner Approach in Health Care: A Conceptual Framework for a Necessary Transition. Acad Med.

[36] Government of Ontario (2000). Ontario Budget 2000.

[37] Ontario Government (2019). A Healthy Ontario: Building a Sustainable Health Care System.

[38] Ontario Government. The Burden of Chronic Diseases in Ontario: Key Estimates to Support Efforts in Prevention. 2019 Jul. Available from: https://www.publichealthontario.ca/-/media/documents/c/2019/cdburden-report.pdf?sc_lang=en. [Cited 2022 Jul 29].

[39] Steffler M, Li Y, Weir S, Shaikh S, Murtada F, Wright JG (2021). Trends in prevalence of chronic disease and multimorbidity in Ontario. Canada CMAJ.

[40] World Health Organization. Preventing Chronic Diseases: A Vital Investment: WHO Global Report. 2005. p. 182. Available from: https://apps.who.int/iris/handle/10665/43314.

[41] Lipscombe LL, Hux JE (2007). Trends in diabetes prevalence, incidence, and mortality in Ontario, Canada 1995–2005: a population-based study. The Lancet.

[42] Diabetes Canada. Diabetes in Ontario: 2022 Backgrounder. 2022 p. 7. Available from: https://www.diabetes.ca/DiabetesCanadaWebsite/media/Advocacy-and-Policy/Backgrounder/2022_Backgrounder_Ontario_English.pdf. [Cited 2022 Jul 29]

[43] Lantz PM (2019). The Medicalization of Population Health: Who Will Stay Upstream?. Milbank Q.

[44] Funnell MM, Anderson RM (2004). Empowerment and Self-Management of Diabetes. Clinical Diabetes.

[45] World Health Organization. Noncommunicable Diseases and Mental Health Cluster. Innovative care for chronic conditions : building blocks for actions: global report. World Health Organization; 2002. Report No.: WHO/MNC/CCH/02.01. Available from: https://apps.who.int/iris/handle/10665/42500. [Cited 2022 May 4].

[46] Jacelon CS, Gibbs MA, Ridgway JV (2016). Computer technology for self-management: a scoping review. J Clin Nurs.

[47] Peeters JM, Wiegers TA, Friele RD (2013). How technology in care at home affects patient self-care and self-management: a scoping review. Int J Environ Res Public Health.

[48] Bitar H, Alismail S (2021). The role of eHealth, telehealth, and telemedicine for chronic disease patients during COVID-19 pandemic: a rapid systematic review. Digit Health.

[49] Kendzerska T, Zhu DT, Gershon AS, Edwards JD, Peixoto C, Robillard R (2021). The effects of the health system response to the COVID-19 pandemic on chronic disease management: a narrative review. Risk Manag Healthc Policy.

[50] Sanchez-Villagomez P, Zurlini C, Wimmer M, Roberts L, Trieu B, McGrath B (2021). Shift to virtual self-management programs During COVID-19: ensuring access and efficacy for older adults. Front Public Health.

[51] Wang H, Yuan X, Wang J, Sun C, Wang G. Telemedicine maybe an effective solution for management of chronic disease during the COVID-19 epidemic. Primary Health Care Research & Development. 2021 ed;22. Available from: http://www.cambridge.org/core/journals/primary-health-care-research-and-development/article/telemedicine-maybe-an-effective-solution-for-management-of-chronic-disease-during-the-covid19-epidemic/431AB4EE6FA683DE3D3633ED9D0F7D99. [Cited 2022 May 5]. 10.1017/S1463423621000517 PMC8488977 34583801

[52] Sabatier PA (1986). Top-down and bottom-up approaches to implementation research: a critical analysis and suggested synthesis. J Pub Pol.

[53] Mcdermott AM, Hamel LM, Steel D, Flood PC, Mkee L (2015). Hybrid healthcare governance for improvement? Combining top-down and bottom-up approaches to public sector regulation. Public Adm.

[54] Government of Ontario. The promotion of healthy living in Ontario: Timeline. Available from: http://www.archives.gov.on.ca/en/explore/online/health_promotion/timeline.aspx. [Cited 2022 Apr 12].

