# Supplementary material for: Evolution of public health policy on healthcare self-management: the case of Ontario, Canada
Source: BMC Health Serv Res. 2023 Mar 14;23:248. doi: 10.1186/s12913-023-09191-3 (PMC10011770; doi:10.1186/s12913-023-09191-3)

**Additional file 1.** Detailed search strategy

1. Archives of Ontario

Three databases that contain archives of the Ontario government were searched: Archives Descriptive database (Ontario), legislative library of Ontario, and the government and legislative libraries online publications (GALLOP) portal

Each search was performed using the search term self-management (archives descriptive database and legislative library of Ontario) or self-management (GALLOP portal). A single search term was used due to the limited possibilities offered by the different portals. For the GALLOP portal, more than 8000 entries were identified using the search term self-management. For this reason, pages were viewed until no new documents could be found (pages screened in total: 20).

Search performed in 2021 (revised November 1, 2021, and May 5, 2022)

1. Government of Ontario website

The government of Ontario website was searched using the search function of the website. A single search term was used due to the limited possibilities offered by the search function of the website. The search term used was self-management. Over 5500 entries were identified using this search term. For this reason, pages were viewed until no new documents could be found (pages screened in total: 10).

Search performed in 2021 (revised November 1, 2021, and May 5, 2022)

1. Database search (Search terms and databases) - Searches performed in 2021 (revised November 1, 2021, and May 5, 2022)


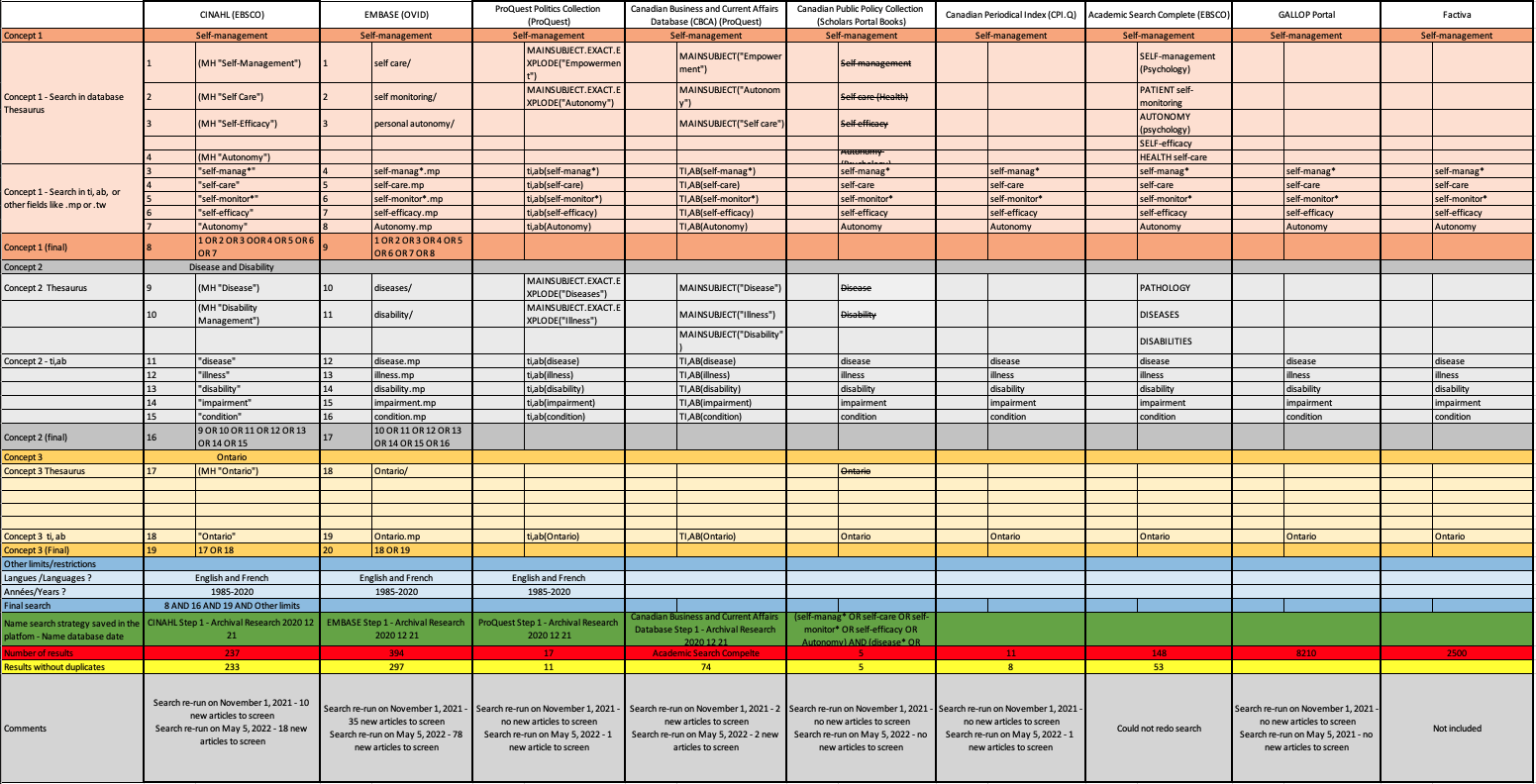

Supplement: Supplementary file 1 — Additional file 1. Detailed search strategy. [file 12913_2023_9191_MOESM1_ESM.docx]
